# A novel fault diagnosis method for second-order bandpass filter circuit based on TQWT-CNN

**DOI:** 10.1371/journal.pone.0291660

**Published:** 2024-02-08

**Authors:** Xinjia Yuan, Yunlong Sheng, Xuye Zhuang, Jiancheng Yin, Siting Yang

**Affiliations:** School of Mechanical Engineering, Shandong University of Technology, Zibo, China; Hainan Normal University, CHINA

## Abstract

To accurately locate faulty components in analog circuits, an analog circuit fault diagnosis method based on Tunable Q-factor Wavelet Transform(TQWT) and Convolutional Neural Network (CNN) is proposed in this paper. Firstly, the Grey Wolf algorithm (GWO) is used to improve the TQWT. The improved TQWT can adaptively determine the parameters Q-factor and decomposition level. Secondly, The signal is decomposed, and single-branch reconstruction is conducted with TQWT to facilitate adequate feature extraction. Thirdly, to capture the time-frequency features in the signal, a CNN-LSTM network is built by combining CNN and LSTM for feature extraction. Finally, CNN, which introduces Fully Convolutional Network (FCN) layers and a Batch Normalization layer, is used to fault diagnosis. The method was comprehensively evaluated with a second-order bandpass filter circuit. The experimental results illustrate that the proposed fault diagnosis method can achieve excellent fault diagnosis accuracy, and the average accuracy is 98.96%.

## Introduction

Analog measurement circuits are significant compositions widely used in measurement circuits, etc [1–3]. The signals measured from sensor measurements must be amplified, shaped, and filtered to extract valuable signals. As an essential part, the filters produce shifts in parameter values with usage and environmental factors such as temperature and humidity, which can affect the test accuracy and make test errors when the parameter values exceed the specified tolerance values [4]. Therefore, it is essential to diagnose faults in the filter circuit in order to ensure test accuracy. Filter circuits can be divided into analog and digital circuits according to their types [5]. Due to the complexity of the circuits themselves, analog circuits are more challenging to diagnose faults than digital circuits. However, the fault rate of analog circuits is four times higher than that of digital circuits [6]. When a fault occurs in an analog circuit, if fault diagnosis methods are not used, it is necessary to detect each test node to determine the faulty component. This method is difficult to identify faulty components and may cause losses to other parts of the circuit during the repair process. Therefore, after setting the tolerance of components, the analog circuit is diagnosed so that the fault components can be located and repaired before affecting the test system’s performance, which is conducive to improving the stability and reliability of the whole test system. Therefore, an urgent need is to study effective analog fault diagnosis methods to locate faulty components accurately.

Filter circuit faults are divided into hard and soft faults. Since soft faults will be transformed into hard faults if they are not handled in time, soft fault diagnosis for analog circuits is one of the effective means to ensure the regular operation of analog circuits.

The existing fault diagnosis methods for analog circuits can be divided into traditional and intelligent fault diagnosis methods. Traditional fault diagnosis methods include the fault dictionary method [7], parameters identification method [8], and fault verification method [9]. These methods require the construction of a mathematical model of the circuit or using simulation software for testing and determining the fault location and type based on the input and output characteristics of the circuit or the component parameter. These methods are computationally intensive and difficult to adapt to circuit structure and parameters. The intelligent fault diagnosis method processes the signal and uses artificial intelligence to classify it for fault diagnosis. In recent years, with the development of artificial intelligence technology, intelligent fault diagnosis methods have also achieved fruitful results.

Since Centeus used wavelet transform to extract the feature parameters of analog circuit fault information [10], Wavelet Packet Transform [11], Wavelet Plus Neural Network [12], and Haar Wavelet [13], which are improved by wavelet transform, have been widely used for the extraction of analog circuit fault features to enhance the accuracy of analog circuit fault diagnosis. Siwek [14] used Support Vector Machine (SVM) to classify LC circuit faults and accomplished accurate analog circuit fault diagnosis localization. Xu [15] used Extreme Learning Machines (ELM) to organize the sample set constructed based on wavelet packet and waveform parameters and completed the fault diagnosis of the elliptic filter. Karthi [16] used Genetic Algorithm(GA) to extract fault information of analog circuits and diagnose them. Subsequently, Mosin [17] used a clustering preprocessing algorithm to extract fault eigenvalues and combined it with a neural network to complete the whole fault diagnosis process, which improved the correct rate of fault diagnosis of analog circuits. Gan [18] first introduced an adaptive factor to enhance the accuracy of the Unscented Kalman Filter (UKF). In order to construct a fault diagnosis model for analog circuits, the improved UKF was used to optimize the parameters of the Wavelet Neural Network(WNN) classifier. Hu [19] proposed a frequency feature extraction method based on the Martingale Distance (MD) to convert the conventional frequency features and frequency response moments into a one-dimensional MD for ana-log filter anomaly detection. Viveros-Wacher and Rayas-Sánchez [20] investigated artificial neural networks for constraint parameter extraction and fault classification for analog fault identification in RF circuits. Shokrolahi [21] used Ensemble Empirical Mode Decomposition (EEMD) for preprocessing of analog circuit fault signals and extracted the fault features from the Intrinsic Mode Functions (IMF) components obtained based on EEMD for composing feature vectors. This method effectively improves the accuracy of analog circuit fault detection and diagnosis. Gan [15] proposed a feature extraction method based on Kernel Supervised Locality Preserving Projection (KSLPP), which combines kernel techniques with supervised locality-preserving projections. Shi [22] proposed a fault diagnosis method combining Density Peaks Clustering(DPC) and Probabilistic Neural Networks(PNN) with dynamic weights that reduces redundant data and achieves high accuracy with a small number of neurons. Aizenberg Igor [23] proposed a multi-valued neural neuron network classifier model, which effectively diagnoses analog circuit faults. Gao [24] proposed a fault diagnosis model for analog circuits based on conditional variational neural networks, which accomplished the fault diagnosis of a variety of filter circuits.

In the above methods, some of the fault data are subjected to feature enhancement, feature extraction, or feature fusion, and then the classifiers are used to classify the faults; some of the data are directly input into the model, and the whole end-to-end fault diagnosis is completed by adaptive feature extraction and a softmax layer. On the one hand, the signal change is relatively feeble when the analog circuit fault occurs, and the feature refinement is significant. At this time, the use of the CNN network end-to-end fault diagnosis method of adaptive feature extraction can not satisfy the requirements of the fault diagnosis; on the other hand, the use of fewer data, faster completion of the fault diagnosis can reduce the loss of the electronic system, and a small amount of data is not conducive to the accurate diagnosis of end-to-end fault diagnosis mode. Inspired by existing fault diagnosis methods [25, 26], this paper proposes a fault diagnosis method based on TQWT-CNN.

Bandpass filters are widely used in measurement systems as devices that filter specific frequencies to obtain power signals at the desired frequency and are an integral part of various electronic systems The use of bandpass filtering circuits can improve the stability of communication and distribution systems, extend the service life of communication and power equipment, and make the distribution system more in line with design specifications for harmonic environments. Therefore using the methodology of this paper the filter circuits are diagnosed for faults. The innovative contributions of the method are as follows:

- This article proposes a fault diagnosis model that can be used for bandpass filter circuits. By combining the improved TQWT with the CNN-LSTM network for feature extraction and the improved CNN for fault recognition, the improved TQWT network can better extract local time-frequency signals with weak features, which is more detailed than the feature extraction of end-to-end models.
- The optimal quality factor Q of TQWT is optimized by using GWO with strong global search ability. The RMSE of the signal with inverse TQWT and the original signal is used as the evaluation criterion to determine the optimal quality factor. The signal is decomposed by TQWT using the optimal quality factor Q, and single-branch reconstruction is performed to complete the decomposition of the signal.
- To improve the classification accuracy and the generalization ability of the model, an improved CNN with FCN and Batch Normalization (BN) layers is built.

## Methods

TQWT is an improvement of Wavelet Transform, which can better adapt to different types of signals and extract data features better and faster. The Grey Wolf Optimizer (GWO) has the advantages of fast convergence, global solid search capability, and robustness. In this paper, GWO is used to optimize the Q-factor of TQWT. CNN-LSTM feature extraction network combines the advantages of CNN and LSTM networks and is widely used for feature extraction. Convolutional Neural Networks(CNN) are characterized by high accuracy in fault diagnosis.

**Fig 1:**
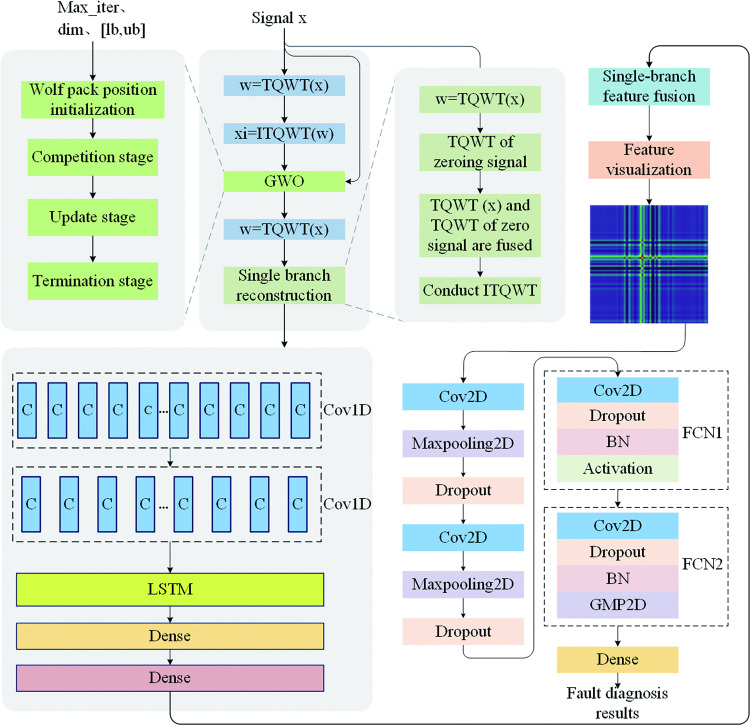
Functional block diagram of the proposed fault diagnosis method.

Given the advantages of each method, the structure for performing analog circuit fault diagnosis is shown in Fig 1. In Fig 1, TQWT (x) is the TQWT transform of the signal x, ITQWT (w) is inverse TQWT of the decomposed signal, and Max_iter, dim, lb, and ub are the number of iterations, dimensionality of the variables, lower bound of the range of searching for the superiority, and the upper bound of the range of searching for the superiority of the GWO, respectively. TQWT and GWO are used for preprocessing the data, the feature extraction method of the data is shown in the CNN-LSTM feature extraction network, and the fault diagnosis technique of the feature dataset is shown in the Fault diagnosis network.

### TQWT

**Fig 2:**
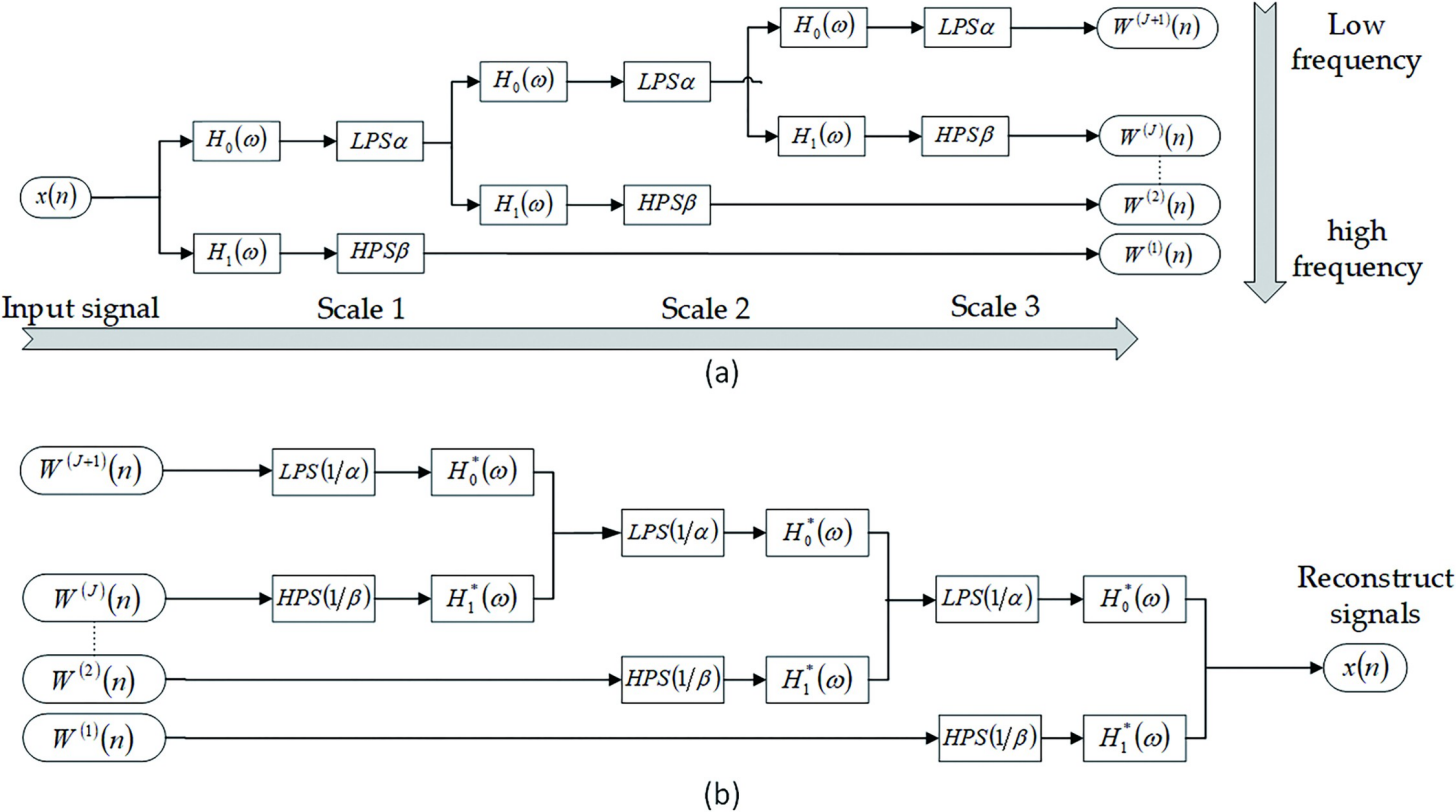
Multiscale TQWT decomposition and reconstruction filter: (a)TQWT signal decomposition process;(b)TQWT signal reconstruction process.

Tunable Q-factor Wavelet Transform (TQWT) is a method for time-frequency analysis and feature extraction of signals [27]; that is, signals are divided into different frequency bands for decomposition according to frequency. Compared with the traditional wavelet transform, TQWT can control the shape of the basis function by changing three key parameters (quality factor Q, redundancy r, and the number of decomposition layers J) according to the characteristics of the signals to be analyzed and realize the decomposition and reconstruction of the signals by means of iterative two-channel filters and discrete Fourier Transforms(FT) in an iterative manner. Adaptive selection of TQWT parameters suitable for the characterization of weak fault features of analog circuits and reconstruction of the subbands obtained from decomposition is essential for analog circuit fault feature extraction. In addition, TQWT decomposes the features of the original signal into local features in different subbands. Compared with the original signal, TQWT provides local refinement of the features, which facilitates subsequent fault diagnosis. A series of iterative two-channel filter banks decompose and reconstruct signals by TQWT. For example, the three-scale decomposition and reconstruction are shown in Fig 2.

In Fig 2, H_0_(ω)、H_1_(ω)are the low-pass and high-pass filter frequency response functions, α、β are the low-pass scale coefficients and high-pass scale coefficients, H_0_^*^(ω)、H_1_^*^(ω) are the corresponding complex conjugates, and ω_j_(j = 1,2,3) represents the transformed wavelet coefficients.

The steps of single-branch reconstruction are as follows: after using TQWT to decompose the signal with length N according to different frequency bands, there are j+1 branches in total. When performing single branch reconstruction of branch i, first perform zero filling interpolation, that is, set the signals of other branches except for branch i to 0, and then perform the inverse transformation of TQWT so as to complete the single branch reconstruction of branch i.

The choice of J only affects the decomposition performance of the TQWT in the low-frequency domain, while Q and r affect the wavelet construction. According to HU [28], the r-value is set to 3. In order to make the TQWT more adaptive to analyze and process the output signal of the circuit, the selection of Q is considered an optimization problem, and the GWO is used to find the optimum for Q iteratively.

In order to make the TQWT more adaptive to analyze and process the output signal of the circuit, the selection of Q is considered an optimization problem, and the GWO is used to find the optimum for Q iteratively. In order to make TQWT more suitable for analyzing and processing the output signal of the circuit, the selection of Q is very important. The decomposition and reconstruction effect of the randomly selected Q value cannot reach the optimal value. Combined with references [29, 30], the selection of Q can be set as an optimization problem, and the optimal value of Q can be found iteratively using GWO. The range of the optimum is set to [1,5]. After determining Q and r, the number of decomposition layers of the TQWT can be determined by Eq (1):

$$J=\lfloor \frac{\text{lg}[N/4(Q+1)]}{\text{lg}(Q+1)/(Q+1-2/r)}\rfloor$$ (1)

In Eq (1), ⌊┤⌋ is rounded to 0, and "N" is the signal length to be analyzed.

The optimized TQWT is used to decompose the signal and obtain j+1 frequency bands according to the frequency; that is, there are j+1 branches from low frequency to high frequency. After single branch reconstruction, the characteristic data of the j+1 group from low frequency to high frequency were obtained. Using TQWT signal decomposition and data single brangle-branching reconstruction, it is suitable for weak feature extraction and provides better local time-frequency signals for subsequent feature extraction, which is conducive to improving the accuracy of the fault diagnosis model.

### GWO

**Fig 3:**
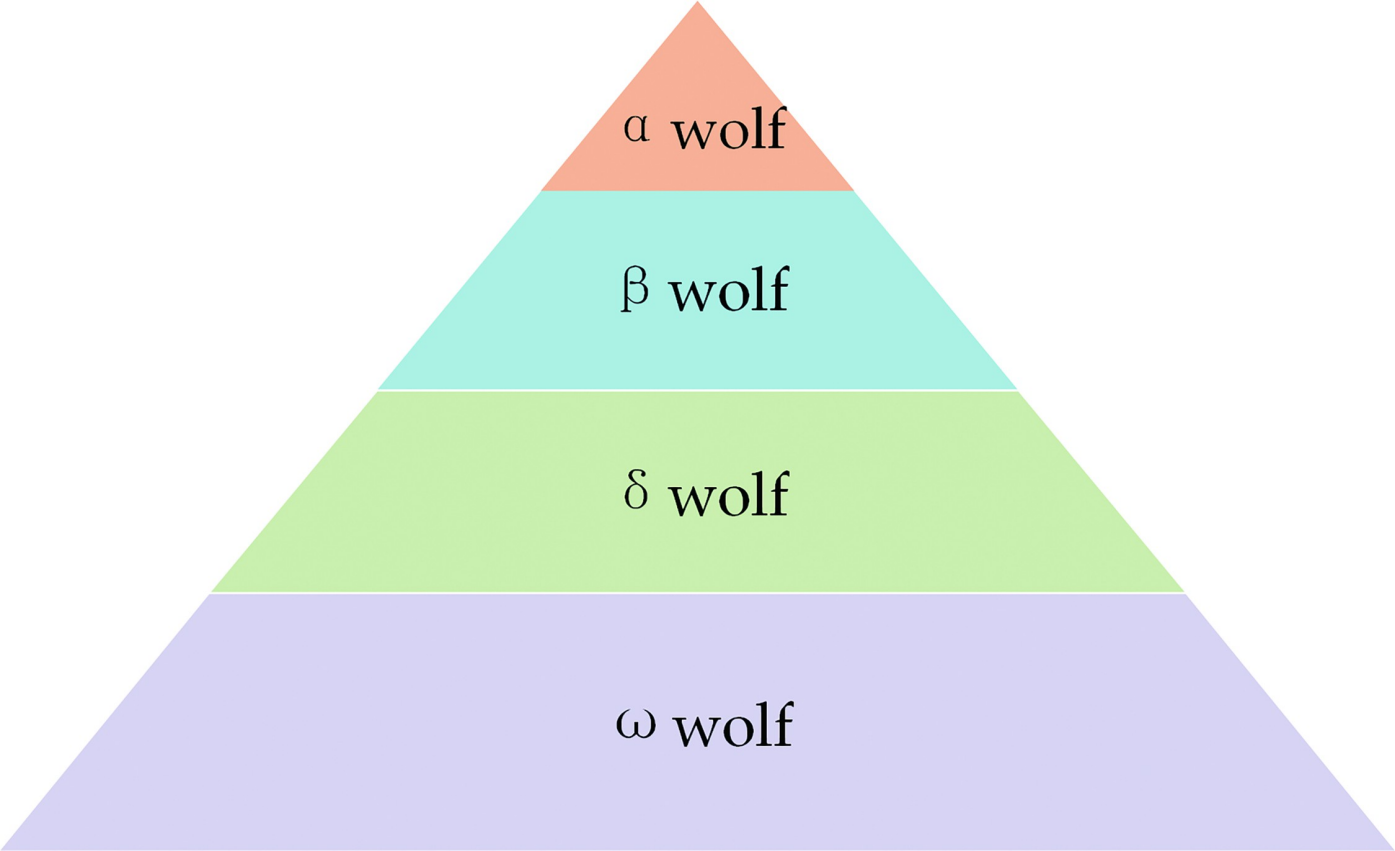
Grey wolf group social rank.

Grey Wolf Optimizer (GWO) is a swarm intelligence optimization algorithm proposed by Mirjalili et al. in 2014 [31]. The GWO finds the optimal solution to the problem by simulating the behavior of grey wolf hunting. In optimization problems, GWO can be used to obtain optimal parameter values within the optimization range, with different optimal parameters that can be obtained by setting different fitness functions. There is a rigorous social ranking system in the grey wolf pack, and the grey wolf ranking in order from highest to lowest is shown in Fig 3 [32]:

The steps for finding the Q-factor of TQWT using GWO are as follows:

#### Initialization stage

Initialize the wolf pack position and calculate the adaptation degree of each wolf by the fitness function. The fitness function is the 1/RMSE of the signal decomposed and reconstructed using TQWT. The equation of the fitness function is shown in equation(2), and the rank of each wolf in the pack is determined according to the fitness.


$$\text{fitness}=1/\sqrt{\frac{1}{\text{n}}\overset{\text{n}}{\underset{\text{i}=1}{\sum }}\;{\left[{\tilde{f}}_{i}-f_{i}\right]}^{2}}$$ (2)

In Eq (2), f_i_ is a point’s function value, ${\tilde{f}}_{i}$ is f_i_ after TQWT and inverse TQWT, and n is the number of data points in the signal data set.

#### Encirclement stage

The grey wolf will gradually encircle the prey during the search process, i.e., the location of the optimal Q-factor, and the mathematical model of the encirclement stage is as follows:

$$\begin{array}{c} D=|C\times X_{P}(t)\;-\;X(t)|\\ X(t+1)=X_{P}(t)\;-\;A\times D\\ A=2ar_{1}-a\\ C=2r_{2} \end{array}$$ (3)

In Eq (3), D represents the distance between the grey wolf and the prey, X represents the position of the grey wolf, t is the number of iterations, XP is the optimal Q-factor position, A and C are coefficient vectors, r1 and r2 are random one-dimensional vectors between [0,^1^], the value of a decrease from 2 to 0 as the number of iterations increases, and a is calculated as shown in Eq (4):

a=2−2×t/T (4)

#### (3) Hunting stage

During the hunting stage, ω wolves are guided by α, β and δ wolves to update their positions. The mathematical model of this behavior is:

$$\left\{\begin{array}{l} {\text{D}}_{\alpha }=|{\text{C}}_{1}{\text{X}}_{\alpha }(\text{t})\;-\;\text{X}(\text{t})|\\ {\text{D}}_{\beta }=|{\text{C}}_{2}{\text{X}}_{\beta }(\text{t})\;-\;\text{X}(\text{t})|\\ {\text{D}}_{\delta }=|{\text{C}}_{3}{\text{X}}_{\delta }(\text{t})\;-\;\text{X}(\text{t})| \end{array}\right.$$ (5)

$$\left\{\begin{array}{l} {\text{X}}_{1}=|{\text{X}}_{\alpha }(\text{t})-{\text{A}}_{1}{\text{D}}_{\alpha }|\\ {\text{X}}_{2}=|{\text{X}}_{\beta }(\text{t})-{\text{A}}_{2}{\text{D}}_{\beta }|\\ {\text{X}}_{3}=|{\text{X}}_{\delta }(\text{t})-{\text{A}}_{3}{\text{D}}_{\delta }| \end{array}\right.$$ (6)

In Eqs (5) and (6), X_α_, X_β,_ and X_δ_ are the positions of wolves α, β and δ, separately.

#### Termination stage

Stop the iteration when the fitness no longer changes or reaches the specified number of iterations, and output the current Q value, i.e., the best Q value obtained from the optimization search.

### CNN-LSTM feature extraction network

**Fig 4:**
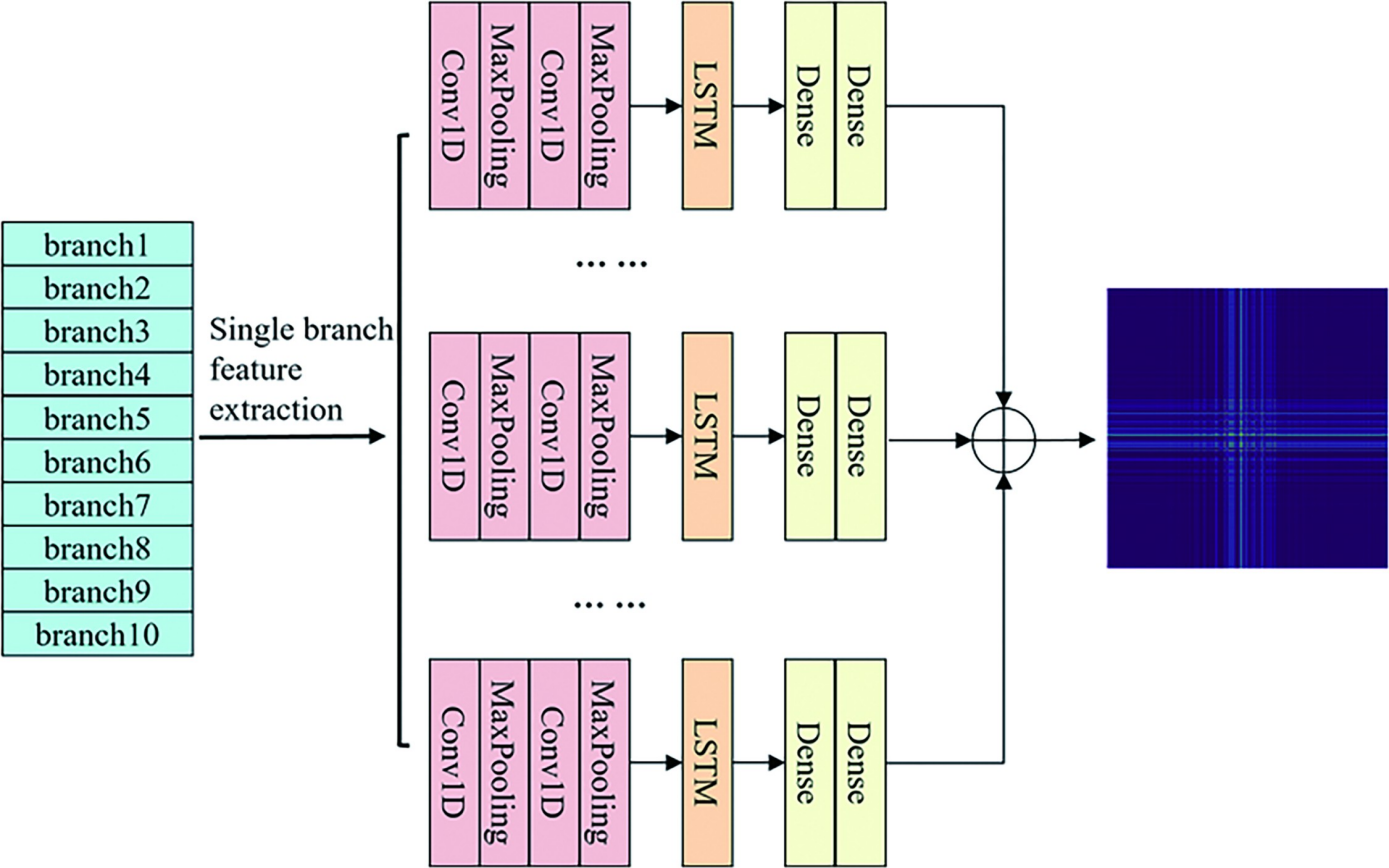
CNN-LSTM network feature extraction process.

CNN-LSTM network is a deep neural network for feature extraction and classification of sequential data, which combines the advantages of Convolutional Neural Network (CNN) and Long Short Term Memory network (LSTM), with CNN layer for capturing local features in the data and LSTM layer for capturing long term dependency relationships in the data [33]. Using CNN-LSTM networks for feature extraction can effectively improve the efficiency of performing feature extraction while reducing overfitting. According to the optimization results and data types, the feature data of 10 branches are obtained after single-branch reconstruction. The CNN-LSTM network is used to perform feature extraction for each branch of the single-branch reconstructed data, and the process of performing feature extraction is shown in Fig 4. In the feature extraction process, the parameters of each layer of the network vary according to the data characteristics to achieve better feature extraction results. Two convolution layer + pooling layer structures are used to improve the feature extraction ability while reducing the risk of overfitting, and then LSTM layers are added to capture the temporal features. To strengthen the model effect, two fully connected layers are finally set. Since the ten branch features are more complex, the data are converted into two-dimensional pictures to input into the fault diagnosis network to avoid losing the relationship between different frequency bands.

### Fault diagnosis network

The fault diagnosis network is based on the traditional CNN, on which FCN and BN layers are used in addition to the convolutional, pooling, and Dropout layers. The FCN layer recovers the class to which each pixel belongs from the abstract features [34], which is more helpful in classifying circuit faults, and the BN layer improves the fault diagnosis network’s generalization ability. The structure diagram of the fault diagnosis network is as follows: two convolution layer + pooling layer structures are used, Dropout layers are added after each pooling structure in order to enrich the training samples, and two FCN layers are built for pixel-level classification of the images, and finally, a Dense layer is added for the output of fault categories.

### Experiment

In order to demonstrate that the method in this paper can solve the problem of diagnosing the faulty components in the filter circuit that deviate from the normal tolerance range caused by the failure of the components, the second-order bandpass filter circuit is selected as the experimental circuit for analog circuit fault diagnosis.

**Fig 5:**
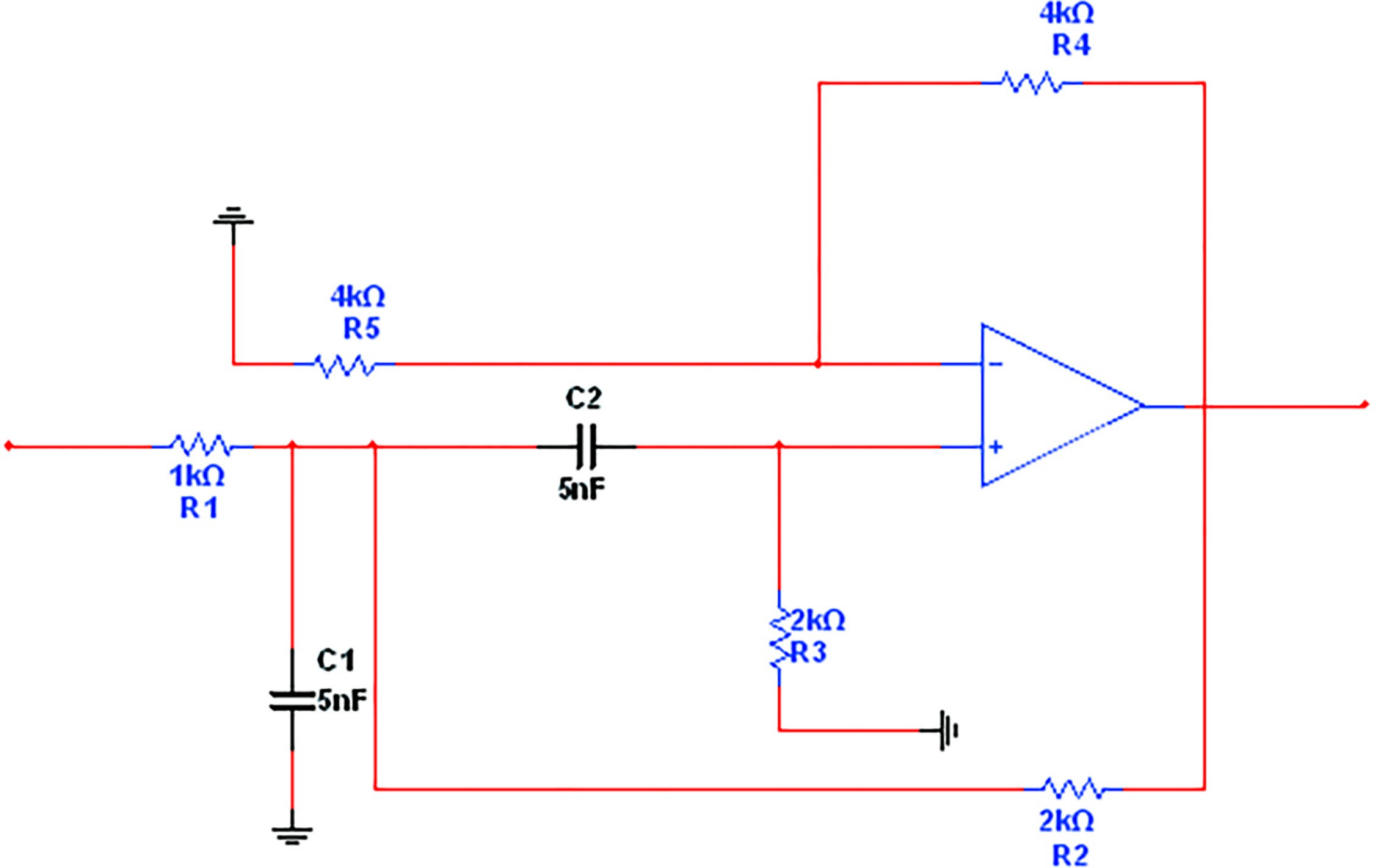
Second-order bandpass filter circuit diagram.

The experiments were conducted on a computer with 16GB RAM, using C++ programming in the Matlab platform to complete the data decomposition, reconstruction, and single-branch reconstruction, and Python programming in the tensorflow environment to complete the rest of the steps. The circuit simulation software is Multisim 14.0. The circuit diagram of the second-order bandpass filter circuit is shown in Fig 5.

### Fault type and fault data acquisition

**Fig 6:**
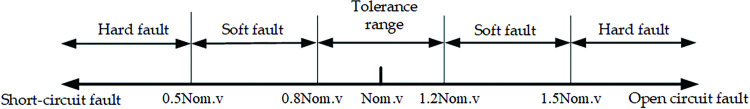
The fault is divided into soft and hard faults.

There are nine representative fault modes in the second-order bandpass filter circuit shown in Fig 6: C1↑, C1↓, C2↑, C2↓, R1↑, R1↓, R3↓, C1↑and R2↓, C1↑and R2↓and R3↓. The ↑ and ↓ represent higher and lower values than the limited range, respectively, and there are nine typical faulty modes and one "faulty-free" mode for ten types of classification. It is assumed that the nominal value of the component is Nom.v. Therefore, the lower limit of soft faults of the component is [0.5Nom.v, 0.8Nom.v], and the upper limit is [1.2Nom.v, 1.5Nom.v]. The fault is divided into soft and hard faults schematically shown in Fig 6, where the nominal values and the component tolerance range of the circuit are shown in Table 1.

**Table 1:** Normal values and faulty range of the second-order bandpass filter circuit.

Fault Class	Fault Tag	Nominal Value	Faulty Range
C1↑	1	5nF	[4nF,6nF]
C1↓	2	5nF	[4nF,6nF]
C2↑	3	5nF	[4nF,6nF]
C2↓	4	5nF	[4nF,6nF]
R1↑	5	1KΩ	[0.8 KΩ,1.2 KΩ]
R1↓	6	1KΩ	[0.8 KΩ,1.2 KΩ]
R3↓	7	2KΩ	[1.6 KΩ,2.4 KΩ]
C1↑and R2↓	8	5nF,2KΩ	[4nF,6nF], [1.6 KΩ,2.4 KΩ]
C1↑and R2↓and R3↓	9	5nF,2KΩ, 2KΩ	[4nF,6nF], [1.6 KΩ,2.4 KΩ] [1.6 KΩ,2.4 KΩ]
Normal	10	/	/

**Fig 7:**
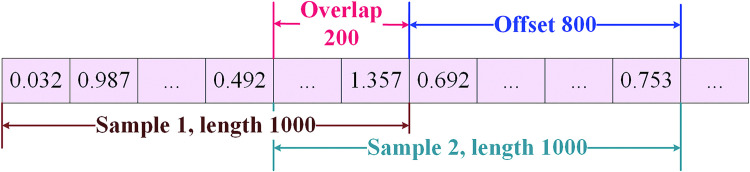
Data sampling method.

Data for each fault type is obtained by keeping the parameters of all components except the component under test within expected values by adjusting the parameters of the component under test to generate fault samples. To enhance the generalization ability of the neural network model, more diverse training samples are used. The data is enhanced by using overlapping sampling [35]. The length of the overlapping part is 200 in order to ensure data diversity, and the overlapping data is sampled in an overlapping manner as shown in Fig 7.

### Structure parameters setting

The entire fault diagnosis process is divided into three parts: data decomposition, feature extraction, and fault diagnosis. The data decomposition stage uses the improved TQWT; the improved method is GWO. When using GWO to optimize Q, its fitness function is RMSE after TQWT decomposition and reconstruction of the signal, as shown in equation (2), the number of species is 1, the number of individuals in the species is 30, the value-seeking range is [1,5], and the number of iterations is 100; in the TQWT, the redundancy degree r is 3, the number of decomposition layers J is 9, and the optimal Q-factor obtained from the search is 1.34. The parameter values are dynamically adjusted according to the data characteristics in the feature extraction stage.

In the fault diagnosis network, two Dense layers are used to improve the capability of nonlinear expression of the network model and its robustness. At this time, in order to improve the generalization ability of the model and break the constraint on the image size of the training and test sets, the Dense layers are replaced with FCN layers, which contain a Convolution layer, a pooling layer, and a Dropout layer. In addition to the FCN layers, one, two, and three Convolution layers are set for fault diagnosis, and 50 independent repeated experiments are conducted to obtain the fault diagnosis accuracy for different numbers of convolution layers as shown in Table 2.

**Table 2:** Accuracy of different convolution layers.

Number of Convolution layers	Accuracy
1	94.25%
2	98.96%
3	97.06%

According to the experimental results, the highest accuracy of fault diagnosis is achieved when two convolutional layers are used, and it is determined that the fault diagnosis network uses two Convolution layers and two FCN layers. The structure and parameters of the fault diagnosis network are shown in Table 3.

**Table 3:** Fault diagnosis network parameters setting.

Layer	Parameters
Conv2D	filters = 512, Strides = (2,2), Kernel_size = (3,3)
MaxPooling2D	pool_size = (3,3), Strides = (2,2)
Dropout	0.2
Conv2D	filters = 128, Strides = (2,2), Kernel_size = (3,3)
MaxPooling2D	pool_size = (2,2), Strides = (2,2)
Dropout	0.2
Conv2D	filters = 64, Strides = (2,2), Kernel_size = (2,2)
Dropout	0.2
Conv2D	filters = 32, Strides = (2,2), Kernel_size = (2,2)
Dropout	0.2
Dense	10

### Experimental results

**Fig 8:**
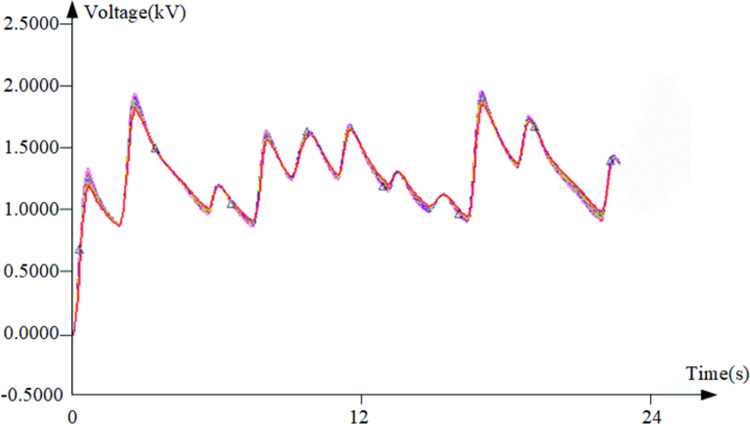
Data curves with fault tag 10.

In the fault diagnosis experiment, considering the complexity of the fault, the sum signal of three sinusoidal signals is used as the excitation source, and the frequencies and amplitudes of the three excitation sources are: "8kHz,10kV", "33kHz,5kV", and "55kHz,3kV". The test point where the test signal was obtained was the output of the circuit. Taking a fault tag of 10 as an example, the data image in the tolerance range is shown in the Fig 8.

**Fig 9:**
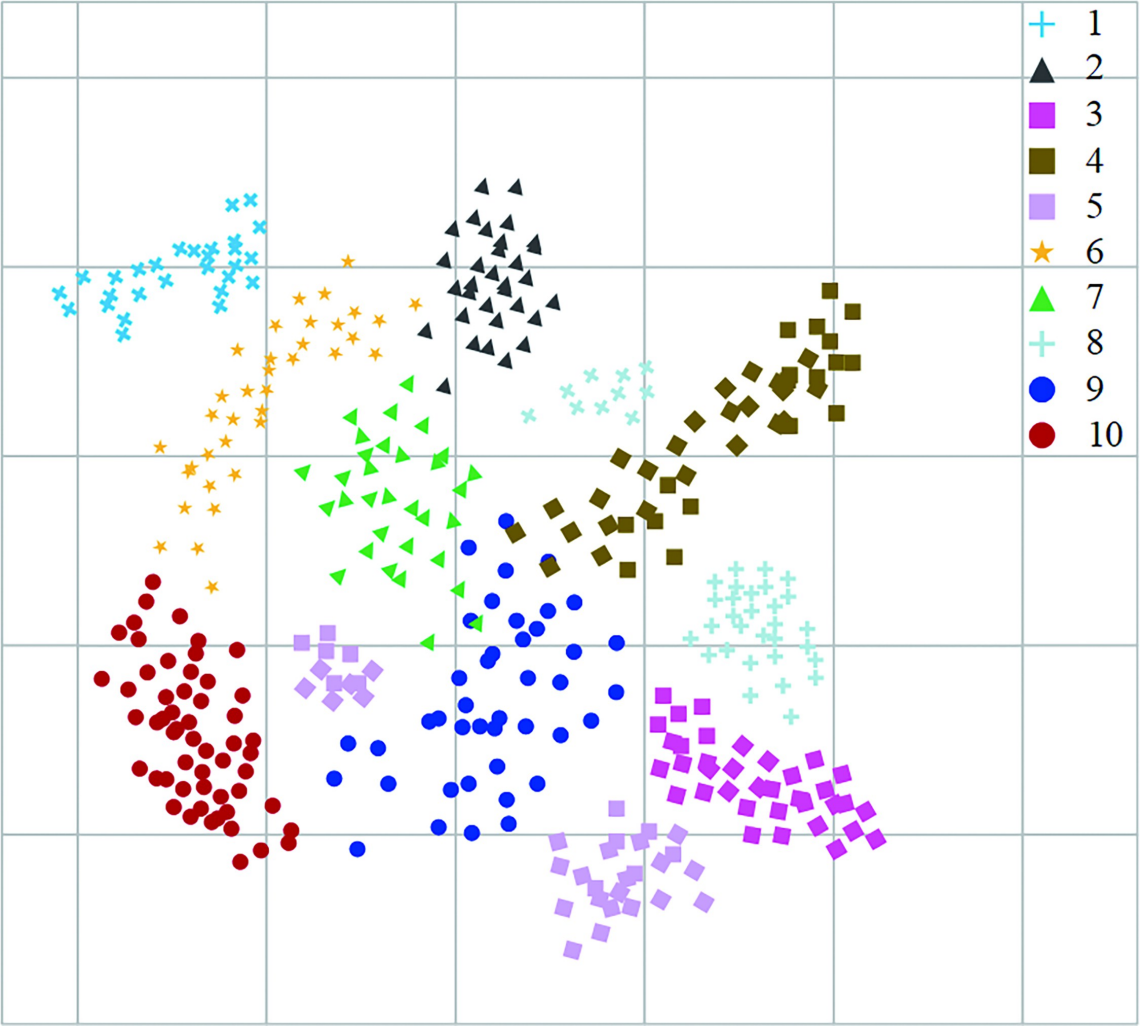
Visualization of the characteristics of the second-order bandpass filter circuit.

According to the fault range, 1295 sets of different types of fault signals were acquired, each containing signals from 1000 acquisition points. The samples were divided into a training set (648 groups) and a test set (647 groups). Fig 9 shows the visualization of the ten fault modes of the second-order bandpass filter circuit by the features extracted by the method in this paper, and it can be seen from the figure that the ten faults that category of the features extracted by the method in this paper can be separated to obtain higher fault diagnosis accuracy.

The confusion matrix represents the fault diagnosis results of the circuit. In the confusion matrix, the column coordinates indicate the actual fault type, the row coordinates indicate the prediction category of the model in this paper, and the values in each column indicate the percentage of accurate predictions to the total number of faults in that category. The evaluation metrics of the confusion matrix are TP (True Positive), TN (True Negative), FP (False Positive), and FN (False Negative). Based on the four evaluation metrics, they are expanded to accuracy, precision, recall, and specificity by combining them with the multi-classification problem. Precision indicates the proportion of samples predicted to be positive classes in the classification model in which the actual category is positive:

$$\text{Precision}=\frac{\text{TP}}{\text{TP}\;+\;\text{FP}}$$ (7)

Accuracy is the percentage of accurate predictions out of the total and is often used to represent model accuracy:

$$\text{Accuracy}=\frac{\text{TP}\;+\;\text{TN}}{\text{TP}\;+\;\text{TN}\;+\;\text{FP}\;+\;\text{FN}}$$ (8)

Recall represents the percentage of the number of positive classes correctly identified by the model as a percentage of the number of truly positive classes:

$$\text{Recall}=\frac{\text{TP}}{\text{TP}\;+\;\text{FN}}$$ (9)

Specificity indicates the proportion of samples predicted to be in the negative category in the classification model for which the correct category is negative:

$$\text{Specificity}=\;\frac{\text{TN}}{\text{TN}\;+\;\text{FP}}$$ (10)

**Fig 10:**
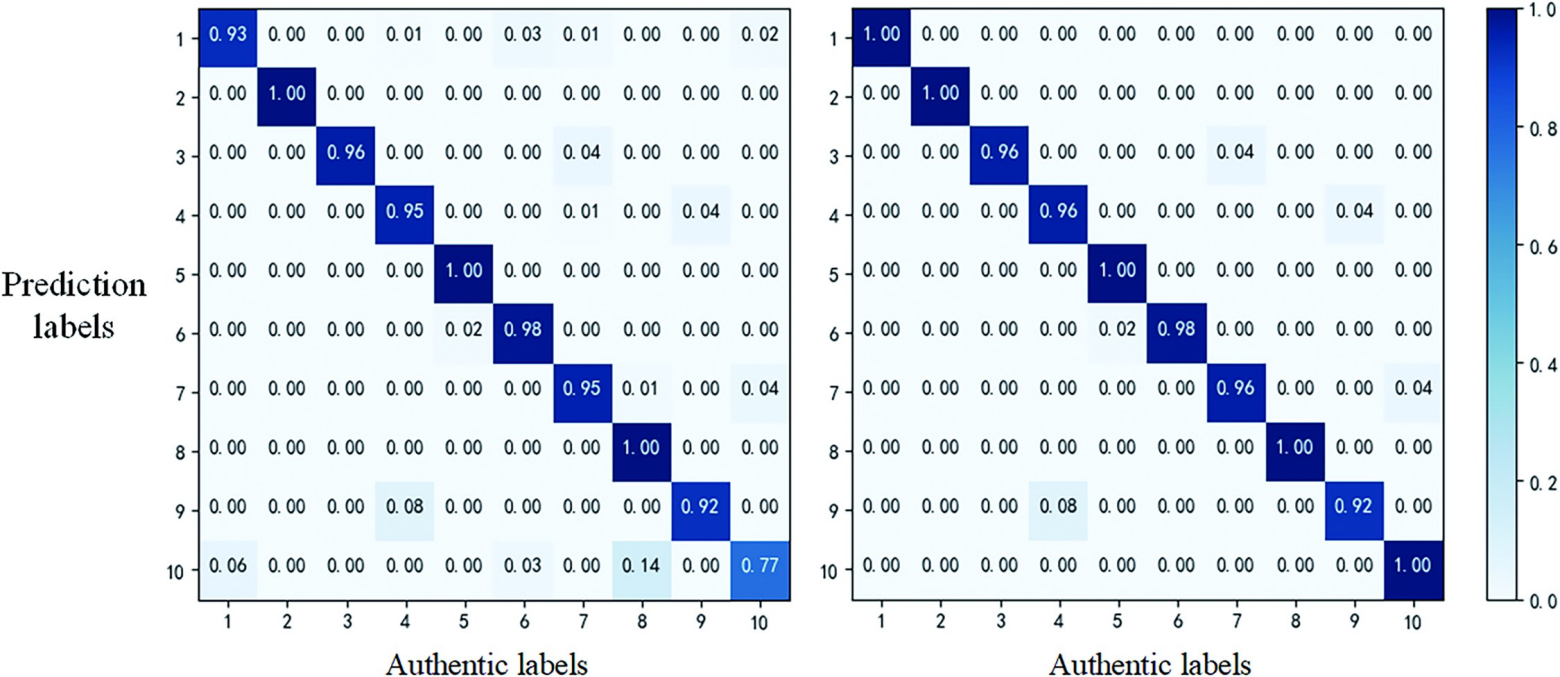
Confusion matrix of fault diagnosis results.

The confusion matrix of the experimental results for fault diagnosis is shown in Fig 10. From the figure, it is clear that the "TQWT-CNN-based fault diagnosis model for second-order bandpass filtered circuits" can achieve fault diagnosis of second-order bandpass filtered circuits. Fifty independent repetitive experiments were conducted on the available data, and the average accuracy was 98.96%.

### Comparison experiments

The fault diagnosis results of the TQWT-CNN based second-order bandpass filtered circuit fault diagnosis model are compared with those of the following models. When using the improved CNN for fault diagnosis, the accuracy is higher than that using SVM for classification due to its network depth with strong fault classification capability; the accuracy using the CNN-LSTM network for time domain feature learning is 5.04% higher than that when no time domain feature extraction is performed; however, the accuracy using only CNN-LSTM for time domain feature extraction also fails to However, the accuracy of using only CNN-LSTM for time-domain feature extraction is also unable to perform fault diagnosis with high accuracy; using GWO for Q-factor optimization of TQWT is also more accurate than using only TQWT for frequency-domain feature extraction for fault diagnosis. From Table 4, it can be seen that this method has a higher accuracy rate, and the operations of signal decomposition, single branch reconstruction, and feature extraction are necessary to improve the accuracy of fault diagnosis of second-order bandpass filter circuits.

**Table 4:** Comparison of fault diagnosis results of different methods.

Method	Accuracy
GWO+TQWT+ Single-branch Reconstruction +CNN-LSTM + improved CNN	98.96%
GWO+ TQWT+ Single-branch Reconstruction+ CNN-LSTM+SVM	87.53%
GWO+ TQWT+ Single-branch Reconfiguration+ Improved CNN	93.92%
CNN-LSTM+ improved CNN	78.50%

The accuracy of comparing the fault diagnosis results of the second-order bandpass filter circuit by comparing the model in this paper with the following models is shown in Table 5. FRFT-CNN for feature extraction using graded Fourier transform to obtain its time-frequency domain features, and it is known from the experimental results that the feature extraction method in this paper extracts more compelling features than FRFT and has a higher fault diagnosis accuracy; the generalized particle swarm algorithm optimized by using The accuracy of feature-based fault diagnosis using a generalized multi-core learning support vector machine optimized by the particle swarm algorithm is 0.59% lower than that of this paper’s model; the accuracy of using an improved empirical modal decomposition method for discriminative feature extraction and feature enhancement of the signal, followed by fault diagnosis using a support vector machine, is slightly lower compared with that of time-frequency domain feature extraction and fault diagnosis.

**Table 5:** Comparison of fault diagnosis results of other different methods.

Method	Accuracy
GWO+TQWT+ Single-branch Reconstruction +CNN-LSTM + improved CNN	98.96%
FRFT-CNN [36]	97.85%
GMKL-SVM [37]	98.37%
IEEMD-SVM [21]	98.69%

The comparison results in Tables 4 and 5 conclude that the model proposed in this paper has good fault diagnosis results. The advantages of using improved TQWT for feature extraction, single-branch reconstruction, and thus better-displaying signal characteristics. Using CNN-LSTM network grouping for feature extraction to make feature extraction more detailed and using improved CNN for better pixel-wise classification of images while preventing overfitting make the model in this paper achieve good results for fault diagnosis of second-order bandpass filter circuits, and the method is reliable and effective.

The local time-frequency data set is obtained by signal decomposition and single branch reconstruction using GWO-optimized TQWT, which is of great significance to the weak fault feature extraction of the data set and is conducive to the accurate fault diagnosis of the fault diagnosis network. In addition, the CNN-LSTM network is used to extract the time-frequency characteristics of the signal, which retains the time-frequency characteristics of the signal, and the obtained features are more sufficient than the end-to-end deep learning fault diagnosis method. Based on this, it is very necessary to carry out data enhancement and local feature analysis in analog circuit fault diagnosis. In addition, fully retaining the time-frequency characteristics of the signal is helpful to the diagnosis of the analog circuit system fault, which can be applied to the research of analog circuit fault diagnosis technology in the future.

## Conclusion

In order to solve the problem of early fault diagnosis of filter circuits, a TQWT-CNN-based fault diagnosis model for analog circuits is proposed. Taking the second-order bandpass filter circuit as a study subject, the GWO-optimized TQWT is used for signal decomposition and single-branch reconstruction, and the CNN-LSTM network is used for time-frequency domain feature extraction after single-branch reconstruction. FCN is introduced based on the CNN network to enhance the model classification ability and BN layer to increase the model generalization ability to complete the fault diagnosis process. The model can effectively identify early faults in second-order bandpass-filtered circuits, and the average recognition rate for identifying and classifying ten fault types is 98.96%. The fault diagnosis method proposed in this paper improves the reliability and stability of the second-order bandpass filter circuit and provides a favorable guarantee for the regular and stable operation of the test system. Future work will focus on applying this fault diagnosis method to actual physical circuits in complex circuits.

## Supporting information

S1 Dataset (ZIP)

## References

[1] Sica M, Tedesco S, Crowe C, Kenny L, Moore K, Timmons S, et al. Continuous home monitoring of Parkinson’s disease using inertial sensors: A systematic review. PLoS One. 2021; 16(2): e0246528. doi: 10.1371/journal.pone.0246528 33539481 PMC7861548

[2] Hubble R P, Naughton G A, Silburn P A, Cole M H. Wearable sensor use for assessing standing balance and walking stability in people with Parkinson’s disease: a systematic review. PloS one. 2015; 10(4): e0123705. doi: 10.1371/journal.pone.0123705 25894561 PMC4403989

[3] Ferreira J, Carvalho E, Ferreira B V, Souza C d, Suhara Y, Pentland A. Driver behavior profiling: An investigation with different smartphone sensors and machine learning. PLoS one. 2017; 12(4): e0174959. doi: 10.1371/journal.pone.0174959 28394925 PMC5386255

[4] Kim Y, Park E, Salim A, Kin J, Lim S. Microwave Dual-Crack Sensor with a High Q-Factor Using the TE20 Resonance of a Complementary Split-Ring Resonator on a Substrate-Integrated Waveguide, Micromachines. 2023; 14(3), 578 doi: 10.3390/mi14030578 36984984 PMC10059035

[5] Zhang C, Ye L, Wu J, Zhang B, Yao N, Wang Y. A Novel Analog Circuit Fault Diagnosis Approach. Recent advances in electrical & electronic engineering. 2021; 14(5): 535–546. doi: 10.2174/2352096514666210713101436

[6] Gao T, Yang J, Jiang S, Yang C. A novel fault diagnostic method for analog circuits using frequency response features, Review of Scientific Instruments. 2019**;** 90(10), 104708. doi: 10.1063/1.5120560

[7] Jia R., Wang J, Zhou J. Fault diagnosis of industrial process based on the optimal parametric t-distributed stochastic neighbor embedding. Science China Information Sciences. 2021; 64, 1–3. doi: 10.1007/s11432-018-9807-7

[8] Marin C.V, Constantinescu F, Nitescu M. A dictionary approach to fault diagnosis of analog circuits. In Proceedings of IEEE, Victoria Falls, Zambia. 2011, 13–15. doi: 10.1109/AFRCON.2011.6072155

[9] Lalami A, Wamkeue R. Synchronous generator off-line diagnosis approach including fault detection and estimation of failures on machine parameters. Electric Power Components and Systems. 2013; 41(15), 1501–1517. doi: 10.1080/15325008.2013.830659

[10] Peng M, Tse C.K, Shen M, Xie K. Fault diagnosis of analog circuits using systematic tests based on data fusion. Circuits, Systems, and Signal Processing. 2013; 32, 525–539. doi: 10.1007/s00034-012-9487-x

[11] Contu S, Fanni A, Marchesi M, Montisci A, Serri A. Wavelet analysis for diagnostic problems. In Proceedings of 8th Mediterranean Electrotechnical Conference, Bari, Italy,. 16–16 May 1996. doi: 10.1109/MELCON.1996.551252

[12] Hong S, Tang J, Chen X, Kong X. Analog circuit fault diagnosis combing wavelet packet with higher order statistics. In Proceedings of 2010 2nd International Conference on Signal Processing Systems, Dalian, China, 05–07 July 2010. doi: 10.1109/ICSPS.2010.5555644

[13] Yang Y, Wang L, Nie X, Wang Y. Incipient fault diagnosis of analog circuits based on wavelet transform and improved deep convolutional neural network. IEICE Electronics Express. 2021; 18(13), 20210174–20210174. doi: 10.1587/ELEX.18.20210174

[14] Siwek K, Osowski S Markiewicz T. Support Vector Machine for Fault Diagnosis in Electrical Circuits. In Proceedings of the 7th Nordic Signal Processing Symposium ‐ NORSIG 2006, Reykjavik, Iceland, 342–345 June 2006. doi: 10.1109/NORSIG.2006.275251

[15] Gan Xu-Sheng, Hong Q, Xiang-Wei M, Chun-Lan Wet. "Research on ELM Soft Fault Diagnosis of Analog Circuit Based on KSLPP Feature Extraction." IEEE Access 7.99(2019):92517–92527. doi: 10.1109/ACCESS.2019.2923242

[16] Karthi S P, Shanthi M Bhuvaneswari M C. Parametric fault diagnosis in analog circuit using genetic algorithm. In Proceedings of 2014 International Conference on Green Computing Communication and Electrical Engineering (ICGCCEE), Coimbatore, India, 06–08 March 2014; 1–5. doi: 10.1109/ICGCCEE.2014.6921410

[17] Mosin S. Quality improvement of analog circuits fault diagnosis based on ANN using clusterization as preprocessing. In Proceedings of 2015 IEEE East-West Design &Test Symposium (EWDTS), Batumi, Georgia, 26–29 September 2015; 1–4. doi: 10.1109/EWDTS.2015.7493158

[18] Gan X, Gao W, Zhe D, Liu W. Research on WNN soft fault diagnosis for analog circuit based on adaptive UKF algorithm. Applied Soft Computing. 2017; 50:252–259. doi: 10.1016/j.asoc.2016.11.012

[19] Hu Z, Xiao M, Zhang L, Liu S, Ge Y. Mahalanobis distance based approach for anomaly detection of analog filters using frequency features and Parzen window density estimation. Journal of Electronic Testing. 2016, 32(6): 681–693. doi: 10.1007/s10836-016-5623-z

[20] Viveros-Wacher A, Rayas-Sánchez J E. Analog fault identification in RF circuits using artificial neural networks and constrained parameter extraction. In Proceedings of 2018 IEEE MTT-S International Conference on Numerical Electromagnetic and Multiphysics Modeling and Optimization (NEMO), Reykjavik, Iceland, 08–10 August 2018; 1–3. doi: 10.1109/NEMO.2018.8503117

[21] Shokrolahi S M, Kazempour A T N. A novel approach for fault detection of analog circuit by using improved EEMD. Analog Integrated. Circuits and Signal Processing. 2019; 98(3):527–534. doi: 10.1007/s10470-018-1362-7

[22] Shi J, Deng Y, Wang Z. Analog circuit fault diagnosis based on density peaks clustering and dynamic weight probabilistic neural network. Neurocomputing, 2020; 407:354–365. doi: 10.1016/j.neucom.2020.04.113

[23] Aizenberg I, Belardi R, Bindi M, Grasso F, Manetti S, Luchetta A, et al. A Neural Network Classifier with Multi-Valued Neurons for Analog Circuit Fault Diagnosis. Electronics, 2021; 10(3):349. doi: 10.3390/electronics10030349

[24] Gao T Y, Yang J L, Jiang S D, Ge Y. A Novel Fault Diagnosis Method for Analog Circuits Based on Conditional Variational Neural Networks. Circuits, Systems, and Signal Processing, 2021; 40:2609–2633. doi: 10.1007/s00034-020-01595-4

[25] Ding Y F, Jia M P, Zhuang J C, Cao Y D, Zhao X L, Chi G L, Deep imbalanced domain adaptation for transfer learning fault diagnosis of bearings under multiple working conditions. Reliability Engineering & System Safety, 2023, 230:108890. doi: 10.1016/j.ress.2022.108890

[26] Han T, Li Y F, Out-of-distribution detection-assisted trustworthy machinery fault diagnosis approach with uncertainty-aware deep ensembles. Reliability Engineering & System Safety, 2022, 226:108648. doi: 10.1016/j.ress.2022.108648

[27] Kumar S A, Subathra M S P, Kumar N M, Malvoni M, Sairamya N J, George T, et al. A Novel Islanding Detection Technique for a Resilient Photovoltaic-Based Distributed Power Generation System Using a Tunable-Q Wavelet Transform and an Artificial Neural Network. Energies, 2020; 13(16): 4238. doi: 10.3390/en13164238

[28] Hu Y T, Zhou Q, Gao J F, Li J, Xu Y G. Compound fault diagnosis of rolling bearings based on improved tunable Q-factor wavelet transform. Measurement Science and Technology, 2021; 32(10):105018. doi: 10.1088/1361-6501/abf25e

[29] Liu X, Sun A, Hu J. Transient feature extraction method based on adaptive TQWT sparse optimization. J Wireless Com Network 2021, 111 (2021). 10.1186/s13638-021-01990-8.

[30] Kong Y, Wang T, Chu F. Adaptive TQWT filter based feature extraction method and its application to detection of repetitive transients. Sci. China Technol. Sci. 61, 1556–1574 (2018). 10.1007/s11431-017-9246-x

[31] Mirjalili S, Mirjalili S M, Lewis A. Grey Wolf Optimizer. Advances in Engineering Software, 2014; 69:46–61. doi: 10.1016/j.advengsoft.2013.12.007

[32] Wang G H, Feng D, Tang W L. Electrical impedance tomography based on grey wolf optimized radial basis function neural network. Micromachines, 2022; 13(7):1120. doi: 10.3390/mi13071120 35888936 PMC9322610

[33] Ye X L, Li M B. Press-fit process fault diagnosis using 1DCNN-LSTM method. Assembly Automation 2022, 2022(3): 42. doi: 10.1108/AA-06-2021-0072

[34] Zhao L, Wang Y, Duan Z X, Chen D F, Liu S P. Multi-Source Fusion Image Semantic Segmentation Model of Generative Adversarial Networks Based on FCN. IEEE Access 2021, 9:101985–101993. doi: 10.1109/ACCESS.2021.3097054

[35] Yu X, Yang J, Xie Z Q. A semantic overlapping community detection algorithm based on field sampling[J].Expert Systems with Applications, 2015, 42(1):366–375. doi: 10.1016/j.eswa.2014.07.009

[36] Song P, He Y, Cui W J. Statistical property feature extraction based on FRFT for fault diagnosis of analog circuits. Analog Integr Circ Sig Process, 2016, 87:427–436. 10.1007/s10470-016-0721-5

[37] Zhang C, He Y, Yuan L, He W, Xiang S, Li Z G. A Novel Approach for Diagnosis of Analog Circuit Fault by Using GMKL-SVM and PSO. J Electron Test, 2016, 32:531–540. 10.1007/s10836-016-5616-y

